# Flail Chest in Polytraumatized Patients: Surgical Fixation Using Stracos Reduces Ventilator Time and Hospital Stay

**DOI:** 10.1155/2015/624723

**Published:** 2015-02-01

**Authors:** Christophe P. M. Jayle, Géraldine Allain, Pierre Ingrand, Leila Laksiri, Emilie Bonnin, Jamil Hajj-Chahine, Olivier Mimoz, Pierre Corbi

**Affiliations:** ^1^Thoracic and Cardiac Surgery Unit, INSERM 1082, Poitiers University Hospital, 2 rue de la Milétrie, 86000 Poitiers, France; ^2^Thoracic and Cardiac Surgery Unit, Poitiers University Hospital, 2 rue de la Milétrie, 86000 Poitiers, France; ^3^Epidemiology & Biostatistics, INSERM CIC0802, Poitiers University Hospital, 2 rue de la Milétrie, 86000 Poitiers, France; ^4^Intensive Care Unit, Poitiers University Hospital, 2 rue de la Milétrie, 86000 Poitiers, France

## Abstract

*Objectives*. Conservative management of patients with flail chest is the treatment of choice. Rib fracture repair is technically challenging; however, with the advent of specially designed molding titanium clips, surgical management has been simplified. Surgical stabilization has been used with good outcomes. We are reporting on our institutional matched-case-control study. *Methods*. Between April 2010 and April 2011, ten polytraumatized patients undergoing rib stabilization for flail chest were matched 1 : 1 to 10 control patients by age ±10 years, sex, neurological or vertebral trauma, abdominal injury, and arm and leg fractures. Surgery was realized in the first 48 hours. *Results*. There were no significant differences between groups for matched data and prognostic scores: injury severity score, revised trauma score, and trauma injury severity score. Ventilator time (142 ± 224 versus 74 ± 125 hours, *P* = 0.026) and overall hospital stay (142 ± 224 versus 74 ± 125 hours, *P* = 0.026) were significantly lower for the surgical group after adjustment on prognostic scores. There was a trend towards shorter ICU stay for operative patients (12.3 ± 8.5 versus 9.0 ± 4.3 days, *P* = 0.076). *Conclusions*. Rib fixation with Stracos is feasible and decreases the length of ventilation and hospital stay. A multicenter randomized study is warranted so as to confirm these results and to evaluate impact on pulmonary function status, pain, and quality of life.

## 1. Introduction

Flail chest has been a devastating injury with high mortality rates ranging from 20 to 30%. In a recent report on flail chest injury published in 2012, mortality was 25%, and flail chest accounted for 9.1% [1]. Patients with severe chest wall injuries develop not only acute morbidities, but also long-term pain and disabilities. Fortunately, most patients with chest wall injuries are successfully treated with analgesics, anti-inflammatory drugs, epidural anesthesia, and aggressive pulmonary toilet. The use of positive pressure ventilation results in improved patient outcomes. However, this technique requires prolonged time on mechanical ventilation, resulting in secondary pulmonary infections and subsequently high mortality rates of 10–36% [1–3]. Moreover, positive pressure ventilation is not always able to reduce and stabilize the bony injury and may even result in painful fracture malunion or symptomatic chest wall deformity.

Operative interventions for flail chest are not frequently performed and are considered by many to be controversial; less than 2% of flail chests are treated surgically [1]. Many of the surgeons who carry out this procedure think that it is underused [4, 5]. Most active academic trauma or thoracic surgeons in the United States have neither performed nor observed a rib fracture operative procedure and are unfamiliar with the literature on surgical indications [6]. Only a few reports on operative repair of severe chest wall injury are associated with low long-term morbidity and pain [4, 7, 8]. There do exist two level one studies comparing operative and nonoperative treatment of flail chest injuries [9, 10]. These two papers demonstrate shorter durations (38% and 78%) of mechanical ventilation, ICU stay, and hospital stay and reduction in mortality due to surgical stabilization.

But rib fracture repair is technically challenging due to the human rib's relatively thin cortex and due to its tendency to fracture obliquely. Nonetheless, several effective repair systems have been developed and applied for use in clinical practice [11, 12]. Two surgical principles for osteosynthesis are available: stabilization with plates and intramedullary stabilization. Stracos molding titanium plate (Strasbourg Costal Osteosynthesis System, MedXpert GmbH, Germany) is a new generation of rib plate with multiple claws that are secondarily tightened to the fractured rib with forceps [13]. Those molded titanium clips simplify plate fixation without screws and reduce the risk of injury to intercostal neurovascular structures. On the other hand, the Judet plate is suitable for spanning a single simple, comminuted, or spiroid fracture. However, no clinical study was done in flail chest patients with this Stracos molding titanium plate. Therefore, we performed a matched-case-control study of treatment of flail chest with this system to evaluate ventilator time and ICU and overall hospital stay.

## 2. Materials and Methods

**Figure 1:**
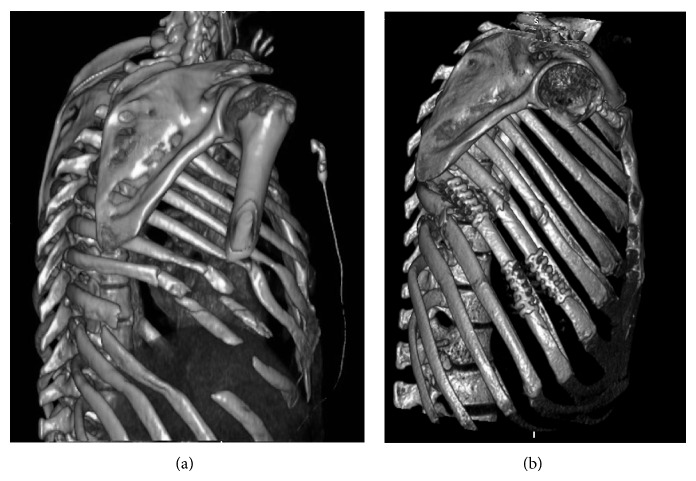
3D-computed tomography bony thorax reconstruction (a) demonstrating flail chest and facilitating the surgical approach and (b) demonstrating flail chest reconstruction, with good cosmetic and functional results.

We conducted a matched case-control study that prospectively included patients with multiple trauma and blunt chest injury that were treated in Poitiers University Hospital in France, from April 2011 to April 2012. All patients with flail chest including bifocal fracture of three or more consecutive ribs in at least two places with or without paradoxical movement underwent rib fixation with Stracos. We excluded patient with aorta hematoma or rupture and patients with tetraplegia or paraplegia and patients having the necessity of neurosurgical treatment. To assess suitability for operation, a 3D-computed tomography bony thorax reconstruction was performed using a 16-multidetector computed tomography scanner (Figure 1(a)). The patients were matched in a 1 : 1 ratio to a control group that did not undergo rib fixation (January 2010 to December 2011, because no fixations were carried out in our institution before January 2011). The matching criteria were as follows: age ± 10 years, sex, number of fractured ribs ± 2, neurological and/or vertebral injury, abdominal injury (spleen, liver, or kidney injury), and arm or leg trauma. Patients with tetraplegia or paraplegia, indication for neurosurgical procedure, aortic rupture, or intramural hematoma, Glasgow < 10, were excluded from this pilot study. These matched patients are chosen retrospectively in the electronic data base of our hospital in accordance with the local ethical committee. This study was accepted by the “Medical Community of Innovation Therapy of Poitiers Hospital.” All patients (or family if necessary) are informed and accepted this new surgical approach before the surgery.

We calculated the injury severity score (ISS), the revised trauma score (RTS), and the trauma injury severity score (TRISS) in all patients. Charts allowed us to collect demographic data, length of intensive care unit (ICU) and hospital stay, ventilation time, and presence of pneumothorax, hemothorax, pulmonary contusion (evaluated on the chest computed tomography (CT) scans), and pneumonia. The number of rib fixations and associated procedures such as running suture of lung parenchyma or suture of diaphragmatic break were noted. In the surgical group, all patients underwent an evaluation of pulmonary function status three months later.

Statistical analysis was performed using SAS for Windows, version 9.2. Quantitative data were presented as mean ± standard deviation (S.D.) and categorical data as percent. Paired samples baseline comparisons used the Wilcoxon test for quantitative variables and the binomial exact test for categorical variables. Outcome variables were normalized by a log-transformation. Univariate analysis used the paired *t*-test. Multivariate analysis by covariance analysis taking into account the matching was adjusted on prognostic scores. At first, we performed the univariate analysis without any difference. Secondarily, we performed a multivariate analysis initially without taking into account the severity scores and then coupled to the severity score. *P* values < 0.05 were considered statistically significant. The study protocol was reviewed and approved by the institutional review board of the Poitiers University Hospital.

### 2.1. Surgical Procedure

The surgical procedure was performed by the same surgeon, always in the first 48 hours after admission. Three-dimensional imaging of the fractured ribs often provided useful information allowing planning the surgical approach. The surgery was performed with general anesthesia. But all these patients, in surgical and medical groups, have an epidural catheter to treat the thoracic pain (performed at the admission in hospital). The patient was placed in the lateral decubitus position. The procedure involved a curvilinear thoracic incision overlying the center of the fractured segments. The hemothorax was drained, and the thoracic cavity was explored. When necessary, we initially performed other procedures such as running suture of lung parenchyma or diaphragmatic suture. The intercostal muscles were dissected off the rib on its superior aspect away from the fracture site, and the fracture was then reduced. We then chose the most suitable Stracos, which is to be found in two available sizes, 6 or 9 claws, according to the length of the fracture. The clip chosen was molded according to the shape of the corresponding rib and the claws were crimped using special pliers on and around the fractured rib.

**Figure 2:**
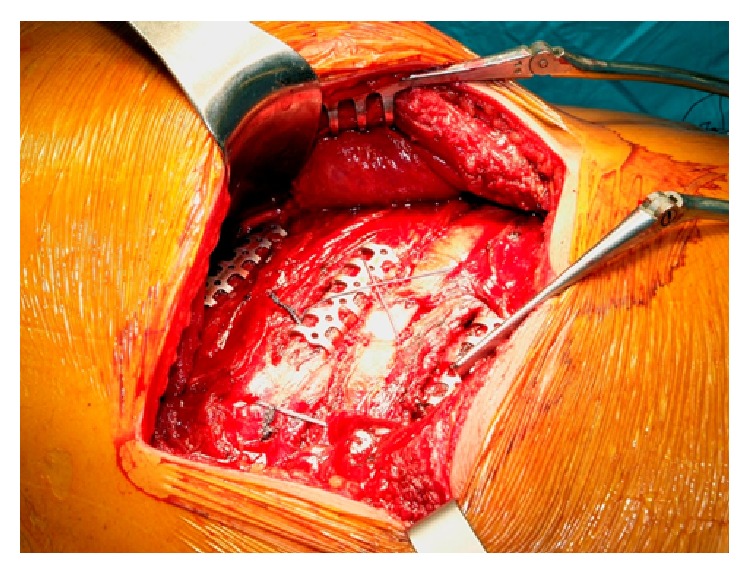
Surgical view after flail chest reconstruction with Stracos. Only one to two ribs were treated with the molding titanium plate, while the other one was stabilized on suture plate.

We treated only one rib out of two with Stracos, and as for displaced or comminuted fractures, the adjacent rib was wrapped using vicryl suture on the osteosynthesis rib (Figure 2). All of the fractures were reduced and stabilized. Two 24 French pleural drains were inserted at the end of the procedure and the wounds were closed, leaving in place a 15 G Blake drain. Mechanical ventilation and drain tube management were conducted according to our institutional protocol.

## 3. Results

The premanagement data of all patients are presented in Table 1. There was no significant difference between the surgical and medical groups. The calculated prognostic scores (ISS, RTS, and TRISS) were similar in both groups. Postmanagement data are summarized in Table 2. Ventilator time and hospital stay were significantly lower for the surgical group after adjustment on prognostic scores. There was a trend towards shorter ICU stay for operative patients. We used 3.3 ± 0.6 molding titanium plates per patient and repaired 3 right diaphragmatic ruptures during surgery. In this case-matched control study, we evaluated the pulmonary function status in the surgery group, and the data are presented in Table 3. In all patients, a 3D-computed tomography bony thorax reconstruction was performed using a 16-multidetector computed tomography scanner three months after surgery (Figure 1(b)).

## 4. Discussion

Our study demonstrates the feasibility of rib fixation for flail chest with Stracos, a new molding titanium plate. This plate greatly simplifies the procedure of flail chest fixation. The plates are used for complex fractures, and we separately treat the fracture without bridging the flail chest. Since they are thin and are manufactured from titanium, they are able to maintain flexibility with respiration movements, thereby limiting the risk of hardware failure secondary to iterative loading. Additionally, the plates are low-profile, prevent hardware irritation, and minimize the need for removal after the fractures heal. When compared in multivariate analysis with matched controls adjusting on prognostic scores, there was a significant difference in ventilator time (142 ± 224 versus 74 ± 125 hours, *P* = 0.026) and in-hospital stay (32.3 ± 19.3 versus 21.7 ± 7.8 days, *P* = 0.024), favoring the surgical group. There was a trend towards shorter ICU stay for operative patients.

Our results are comparable to those of other reports. In a retrospective case-control study, Nirula et al. showed a decrease in postoperative duration on mechanical ventilation in patients who underwent stabilization of their rib fractures after blunt chest injury [7]. In the first randomized study for flail chest treatment, Tanaka, who stabilizes flail chest with Judet struts, showed that the surgically treated group demonstrated a significantly shorter ventilator duration and ICU stay, along with lower incidence of pneumonia. In the second prospective study by Granetzny et al., the group that received surgical treatment also presented significant reduction in ventilation days and shorter hospital and ICU stay compared with the conservatively treated group [9].

In the present study, the number of patients was small, but the difference was significant. This was certainly due to the fact that surgery was performed in the first 48 hours after the admission. Rapid intervention is a topic of discussion with regard to the different case-control studies on flail chest fixation. The longer the surgery is delayed, the greater the length of ICU or hospital stay is, and the difference between the two groups decreases. Many experts also believe that the underlying lung injury, rather than the bone injury, is the major contributor to the morbidity and mortality following flail chest injuries: Voggenreiter et al. attempted to address this issue by retrospectively comparing the outcomes of surgical fixation of the flail segment in patients with or without underlying pulmonary contusion [14]. They also evaluated 18 matched patients without pulmonary contusion treated nonsurgically. Fractures were fixed with Judet struts or 3.5 mm plates. They found significant differences in the duration of mechanical ventilation (30.8 versus 6.5 days), rates of pneumonia (40% versus 10%), and mortality (30% versus 0%) between the surgical patients with and without pulmonary contusion. They also found significant differences in the duration of ventilation (6.5 versus 26.7 days) and pneumonia (10% versus 27%) between the surgical and nonsurgical patients without pulmonary contusion. They suggested that patients with flail chest and no contusion have better outcomes if surgical stabilization is performed early after admission, while patients with flail chest and pulmonary contusion should be fixed only if paradoxical motion or progressive collapse is noted. In our series, timing of surgery was always before the 48th hour with or without pulmonary contusion diagnosed on the CT scan, but all patients were in fact victims of severe trauma with paradoxical motion as shown by the number of ribs fractured (6.6 ± 2.9 versus 7.7 ± 2.4, *P* = 0.39). As was the case with De Moya et al., we consider this to be an important topic of discussion in the framework of another, larger randomized multi-institutional trial [15].

This study has several limitations as a case-controlled study. The small sample size decreases the power of the statistical comparisons and may account for a type II error. In our study, it was also necessary to adjust prognostic scores (ISS, RTS, and TRISS) so as to obtain significant differences in the outcomes. A case-controlled study with nonrandomized historical controls limits its level of evidence; it is therefore necessary to confirm results with a randomized trial. However, a retrospective study seemed to be the most feasible starting point in evaluation of a new molding titanium plate. Given the very low incidence of the rib fixation procedure in trauma centers and the absence of data from previous studies on the plate requirements involving patients with rib fractures, this pilot study was necessary in order to determine a power analysis for a randomized trial.

After this pilot study, we are conducting a randomized trial comparing surgical rib fixation with Stracos and medical care. Having a control group one year before the institution of surgical treatment certainly represents another bias. Medical care protocols remained unchanged for two years; management of pain and ventilation were likewise the same always with epidural catheter. Moreover, during the year of surgical treatment, all patients with flail chest underwent surgical rib stabilization. If we determine a control group in the same year, all patients who have no indication or a contraindication of rib fixation will constitute the latter group, and this will be a major source of bias.

Since this is a pilot study, long-term follow-up is lacking. We studied functional pulmonary status by pulmonary function tests in the 10 patients three months after surgery. Retrospectively, we were not able to study this data in the control group. In the surgical group, forced vital capacity (FCV) and total lung capacity (TLC) were superior to 75% in 90% of the cases. These excellent results are in normal ranges with no sequelae on the pulmonary function. In his randomized trial, Granetzy noted significantly improved pulmonary function tests performed at 2 months after injury in the operative group [9]. In 2009, Mayberry et al. also stated that operative repair of severe chest wall injuries is associated with low long-term morbidity and pain, as well as health status nearly equivalent to the general population [6].

Finally, we know that surgery is an expensive option procedure, especially with the titanium material. Nevertheless, Tanaka and colleagues reported that the total medical expense in their small, randomized, non-US trial was almost $10,000 lower per patient in the operative group compared with those treated with internal pneumatic stabilization [10]. Bhatnagar et al. also showed that flail chest surgery is a more cost effective strategy than standard of care, and it remained the most cost effective strategy throughout the range of ventilator-associated pneumonia and the quality of life rates [5].

In conclusion, the indications for fixation of flail chest injuries remain controversial primarily on account of a lack of adequate studies comparing operative and nonoperative treatment. However, several studies suggest a substantial benefit in patients with flail chest injuries requiring mechanical ventilation which undergo surgical stabilization. Despite the evidence reported therein, the procedure generally remains underutilized. The use of molding titanium plates greatly simplifies the procedure of flail chest fixation. The plates are used for complex fractures, and we separately treat fracture(s) without bridging the flail chest. In this pilot study, rib fixation significantly decreases ventilation time and hospital stay. This work will help us to conduct, as of 2014, a multicenter randomized prospective study in France, comparing outcomes, quality of life, pulmonary function, return to work, and total cost of care.

## Figures and Tables

**Table 1:** Premanagement data of the 10 patients with flail chest on admission and paired matched patients.

	Medical group	Surgical group	*P* value (paired sample)
Sex ratio (*n* males, %)^(1)^	8 80%	8 80%	1
Age^(1)^	50.5 ± 12.5	47.9 ± 10.6	0.48
Number of ribs fractured^(1)^	6.6 ± 2.9	7.7 ± 2.4	0.39
Abdominal injury (*n*, %)^(1)^	2 20%	2 20%	1
Neurological injury (*n*, %)^(1)^	5 50%	5 50%	1
Vertebral injury^(1)^	4 40%	6 60%	0.69
Hemothorax (*n*, %)	7 70%	8 80%	1
Pneumothorax (*n*, %)	8 80%	8 80%	1
Pulmonary contusion	9 90%	8 80%	1
Arm trauma^(1)^	6 60%	6 60%	1
Limb trauma^(1)^	3 30%	2 20%	1
Glasgow score	14.4 ± 1.6	14.0 ± 2.1	1
ISS	26.1 ± 6.2	28.6 ± 8.7	0.41
RTS	7.7 ± 0.2	7.3 ± 0.7	0.12
TRISS	7.6 ± 7.1	11.4 ± 15.3	1

ISS: injury severity score, RTS: revised trauma score, and TRISS: trauma injury severity score.

^(1)^Matching criteria.

**Table 2:** Postmanagement data in 20 patients.

	Medical group (*n* = 10)	Surgical group (*n* = 10)	*P* in univariate analysis	*P* in multivariate analysis^(1)^
ICU stay (days)	12.3 ± 8.5	9.0 ± 4.3	0.421	0.076
Ventilator time (hours)	141.6 ± 224.4	73.5 ± 124.7	0.699	0.026^∗^
Total hospital stay (days)	32.3 ± 19.3	21.7 ± 7.8	0.250	0.024^∗^
Pulmonary infection (*n*, %)	3 30%	4 40%	1	
Number of Stracos	—	3.3 ± 0.6 [2–6]	—	
Diaphragmatic break	—	3	—	

^(1)^Multivariate analysis by analysis of covariance adjusted on prognosis factors (ISS, RTS, and TRISS) and taking into account the matching. ^∗^Significant *P* value.

**Table 3:** Pulmonary function tests in the 10 patients, three months after surgery.

	Means ± SD	*n* > 75%
FVC	90.2 ± 13.2	9
FEV_1_	77.6 ± 12.1	7
TLC	93.1 ± 7.6	9
PEFR	92.2 ± 2.2	10

Forced vital capacity (FVC), forced expiratory volume in the first second (FEV_1_), total lung capacity (TLC), and peak expiratory flow rates (PEF).
